# Review of the relationship and underlying mechanisms between the Qinghai–Tibet plateau and host intestinal flora

**DOI:** 10.3389/fmicb.2022.1055632

**Published:** 2022-11-29

**Authors:** Jin Lv, Ping Qi, Liu-Hui Bai, Xiang-Dong Yan, Lei Zhang

**Affiliations:** ^1^The First Clinical Medical College, Lanzhou University, Lanzhou, China; ^2^Department of General Surgery, The First Hospital of Lanzhou University, Lanzhou, China; ^3^Key Laboratory of Biotherapy and Regenerative Medicine of Gansu Province, The First Hospital of Lanzhou University, Lanzhou, China

**Keywords:** Qinghai-Tibet plateau, intestinal flora, hypoxia, cold climate, high altitude

## Abstract

The intestinal microbial community is the largest ecosystem in the human body, in which the intestinal flora plays a dominant role and has a wide range of biological functions. However, it is vulnerable to a variety of factors, and exposure to extreme environments at high altitudes, as seen on the Qinghai–Tibet plateau, may cause changes in the structure and function of the host intestinal flora. Conversely, the intestinal flora can help the host adapt to the plateau environment through a variety of ways. Herein, we review the relationship and underlying mechanism between the host intestinal flora and the plateau environment by discussing the characteristics of the plateau environment, its influence on the intestinal flora, and the important role of the intestinal flora in host adaptation to the plateau environment. This review aimed to provide a reference for maintaining the health of the plateau population.

## Introduction

The structure and function of intestinal microorganisms has been well studied, with most studies focusing on intestinal bacteria. This intestinal flora contains many types of bacteria, many of which are closely associated with human health (Schroeder and Backhed, 2016; Barko et al., 2018). High-altitude regions have low oxygen, extreme cold temperatures, and high radiation levels, all of which having a profound impact on the structure and function of the intestinal flora. The Qinghai–Tibet Plateau in China is the largest plateau area in the world, and visitors experience symptoms of discomfort, especially headache, chest tightness, nausea, and vomiting (Wen et al., 2014; Croughs et al., 2022). Kleessen et al. (2005) reported that dysbiosis, in which pathogenic bacteria increase and probiotics decrease, often occurs in plateau populations. This dysbiosis can lead to the translocation of bacteria and endotoxins to the blood, triggering sepsis, multiple organ dysfunction syndrome, and other diseases (Wang, 2021). A comprehensive and in-depth understanding of the effects and mechanisms of high-altitude conditions on human intestinal flora is particularly important to maintain the health of high-altitude populations. However, to the best of our knowledge, few relevant studies have been published leading to poor understanding of the relationship between the intestinal flora and plateau environment and the underlying mechanisms and their effect on host health. This paper focuses on the structure and function of intestinal flora in humans and some mammals at high altitudes, which hopefully will lead to further studies on this topic, and we hope that our review will contribute to the development of methods to alter the intestinal flora to improve the health of people at high altitudes.

## Qinghai–Tibet plateau

A flat landform elevated 3,000 meters above sea level is called a plateau. The air pressure in plateau areas is only 50% of that in plain areas, and the boiling point of water is below 90^°^C. With the increase in altitude, air oxygen content decreases; in areas elevated more than 3,000 and 5,500 m above sea level, air oxygen content decreases by 30 and 50%, respectively. Plateau regions generally have relatively low temperatures, with large temperature differences between day and night. For example, the Qinghai–Tibet plateau, which is the highest plateau in the world and is known as the “roof of the world” or the “Asian water tower,” has an average annual daily temperature of 3.1^°^C (Xu et al., 2011). The sunshine duration is long in the plateau region, and ultraviolet radiation is much higher than that in the low-altitude area. The population of the Qinghai–Tibet plateau maintains their traditional grazing diet, comprising beef and mutton, dairy products, and less fruit and vegetables, and their vitamin C consumption is much lower than that of the plain population (Lan et al., 2017). Their daily staple food is “Zanba,” consisting of plateau barley as the main raw material, and their common drink is butter tea (Moore et al., 1998).

The low oxygen concentration at high altitudes can inhibit aerobic metabolism, and the insufficient energy supply results in declining circulatory function in the body and a corresponding reduction in nutrients and energy supply levels in tissues and organs. In studies on high-altitude immigrants, to compensate for oxygen deficiency, increases in heart rate and cardiac output, red blood cell count and volume, or hemoglobin concentration are often observed (Moore et al., 1998). In decompensation, hypoxemia may develop into severe altitude sickness (Luks, 2015). Hypoxia can reduce the activity of Na^+^/K^+^-ATPase in the cell membrane, resulting in energy metabolism disorders in brain cells, which leads to high-altitude cerebral edema (Sun et al., 2002). Furthermore, acute exposure to high altitudes can increase capillary pressure and hypoxic pulmonary vasoconstriction, leading to increased pulmonary artery pressure and high-altitude pulmonary edema (Maggiorini et al., 2001).

Despite the negative impacts of living at high altitudes, people who live on the Qinghai–Tibet plateau have adapted to the plateau environment. They have a high vagal tone, and the heart rate and heart rate variability of plateau adolescents are lower than those of the plain adolescents. The lungs of individuals in the plateau population grow faster during adolescence, and the lung capacity and volume of adults are larger than those of the plain population, increasing the surface area of gas exchange (Weitz et al., 2016). The density of capillaries in the muscles of individuals in the plateau population is higher, which leads to improved perfusion and oxygen transport and higher blood flow. These factors may counteract the effects of the low arterial oxygen levels in the plateau population (Hoppeler et al., 2003; Beall, 2007). In addition, the high nitric oxide (NO) content, which is synthesized as a vasodilator in the inner blood vessel walls, might play a role in the pulmonary hypotension seen in the Qinghai–Tibet plateau population (Weitz et al., 2016). Genetic variation at the *MTHFR* locus, encoding a key enzyme for folic acid metabolism, is associated with increased folic acid levels, which helps Tibetans resist high-dose ultraviolet radiation (Yang et al., 2017). Highly expressed *HMOX2* in Tibetans is involved in heme catabolism, and *HMOX2* contributes to high-altitude adaptation by serving as a regulator of hemoglobin metabolism (Yang et al., 2016). Genes, such as *EPAS1*, *EGLN1*, and *PPARA*, have also been reported to help Tibetans adapt better to the harsh hypoxic environments (Simonson et al., 2010; Peng et al., 2011).

## Factors affecting the intestinal flora in the Qinghai–Tibet plateau population

### Hypoxia

Hypoxia can damage organs, tissues, and cells and is involved in the occurrence of various diseases (Pasha and Newman, 2010; Taylor and Colgan, 2017). Furthermore, a steady state of oxygen content in the intestine is crucial for maintaining the composition and function of the intestinal flora (He et al., 1999). After 12 days of exposure to a hypoxic environment, the Firmicutes/Bacteroidetes ratio and alpha diversity index decreased in the intestinal flora of guinea pigs (Lucking et al., 2018). In addition, after exposing mice to intermittent oxygen for 6 weeks, Firmicutes in the intestinal tract increased, Bacteroides and Proteobacteria decreased, and the alpha diversity index increased compared with those in the control group (Moreno-Indias et al., 2015).

Typically, anaerobes dominate the intestinal tract of their host (Suzuki et al., 2019). Under hypoxic conditions, anaerobes in the host intestine become more competitive and overgrow (Moreno-Indias et al., 2015). Suzuki et al. (2019) found a positive correlation between obligate anaerobes and altitude, and a negative correlation between facultative anaerobes, aerobic bacteria, and aerobic bacteria and altitude. Adak et al. (2013) found that, 15 days after reaching 3,505 m above mean sea level in a plateau area, the amount of aerobic bacteria in the intestine of stationed soldiers reduces 50-fold, while the amount of anaerobic bacteria increases 115-fold. Furthermore, compared to that in the normal oxygen control group, the intestinal flora of rats in a hypoxic environment had a decreased amount of aerobic bacteria and an increased amount of obligate anaerobes (Maity et al., 2013).

Hypoxia in patients with lung disease often leads to changes in the structure of the intestinal flora. For example, changes in the diversity and abundance of the intestinal flora in patients with viral pneumonia were observed (Groves et al., 2018). Patients with chronic obstructive pulmonary disease are deprived of oxygen for extended periods because of chronic dyspnea, and according to a survey, 40.26% of patients with chronic obstructive pulmonary diseases have an imbalance in their intestinal flora (Huang et al., 2017). Moreno-Indias et al. (2015) observed similar changes in the intestines of mouse models that mimic sleep apnea syndrome.

### Cold environment

Cold is an important factor that affects the structure of the intestinal flora (Chevalier et al., 2015). Exposure to cold environments can reduce the abundance of the intestinal flora. The abundance of intestinal flora decreased after being soaked in cold water for 1 h (Zhang et al., 2021). Furthermore, Zietak et al. (2016) housed mice in a cold environment and observed a decrease in the abundance of their intestinal flora.

In one study, mice were randomly divided into two groups: the experimental group was housed at 5^°^C and the control group was housed at 21^°^C. After 1 week, the intestinal *Enterobacter* and *Enterococcus* levels in the experimental group were more abundant than those in the control group, whereas the abundance of Bacteroides *Lactobacillus* and *Bifidobacterium* decreased. In the intestinal tract of mice exposed to a cold environment, the phylum Firmicutes increased, the abundance of Bacteroides decreased, and the phylum Verrucomicrobia almost disappeared (Chevalier et al., 2015). Zheng et al. (2020) housed pregnant rats on the 14th day of gestation in a cold environment until delivery and found that the abundance of Bacteroides *Lactobacilli* in the intestinal tract of the offspring increased, whereas the abundance of *Prevotella* decreased.

### Ultraviolet radiation

Excessive exposure of the skin to ultraviolet radiation can affect the structure of the host intestinal flora. After three rounds of ultraviolet radiation in a week, the alpha and beta diversities of bacteria in the intestinal tract of females increased, and the number of multiple bacterial genera changed (Bosman et al., 2019). Furthermore, excessive ultraviolet radiation to the human body can lead to the emergence of intestinal flora in the thick wall of the phylum, increased deformation of the phylum, and a reduction in Bacteroides (Conteville and Vicente, 2020). Ghaly et al. (2018) found similar changes in the intestinal tract of mice exposed to high levels of ultraviolet light.

## Changes in the intestinal flora of the Qinghai–Tibet plateau population

The Qinghai–Tibet plateau environment can influence changes in host oral and skin microbiota and affects and shapes the intestinal flora (Li et al., 2019; Liu F. et al., 2021; Table 1). The abundance of bacteria in the intestinal tract of people living in the plains considerably decreases a few years after moving from a low to high altitude (Li and Zhao, 2015). A high-altitude environment can increase the abundance of pathogenic bacteria in the host intestine. Li et al. (2016b) found that the abundance of pathogenic bacteria, such as *Enterobacter* and *Proteus*, in the intestinal tract of plain populations that migrated to the plateau was higher than that of the plain populations who did not migrate. In the intestines of seven soldiers situated in the Himalayas at an altitude higher than 5,000 m, the abundance of potential pathogenic gram-negative bacteria, especially of the Enterobacteriaceae family, increased, while the abundance of probiotics, such as *Bifidobacteria*, decreased. All these individuals had different degrees of maladaptive symptoms, such as diarrhea and infection (Kleessen et al., 2005). This is slightly different from the results of an animal experiment by Adak et al. (2014) in which male rats were exposed to a simulated high altitude for 30 d. Although the abundance of *Escherichia coli* in the intestinal tract increased 125-fold, *Bifidobacterium* and *Lactobacillus* also increased several folds. This environment also caused anorexia in rats, resulting in weight loss, negative nitrogen balance, intestinal barrier dysfunction, and mucosal injury, thus enhancing bacterial translocation and inducing inflammation (Adak et al., 2014). Pan et al. (2022) reported increases in *Parabacteroides*, *Alistipes*, and *Lactococcus* genera and an increase in *Bacteroides*-to- *Prevotella* ratio in rat intestinal tract under simulated high-altitude environment. During this period, the rats ate less, their body weights decreased sharply, and pathological myocardial hypertrophy occurred. After rapidly entering the plateau environment at an altitude of 4,100 m, rats had markedly increased intestinal *Bacteroides* and markedly decreased *Corynebacterium*, *Preceptella*, and *Coprococcus* populations; the metabolic activities of the flora were also weakened.

**TABLE 1:** Changes in the intestinal flora of the Qinghai–Tibet plateau population.

Experimental subjects	Phylum	Family	Genus or order	References	Sequencing method
People	Firmicutes ↑		*Acidaminococcus*, *Actinomyces*, *Blautia*, *Butyricimonas*, *Clostridium*, *Desulfovibrio*, *Helicobacter*, *Leuconostoc*, Peptostreptococcaceae Prevotellaceae uncultured, *Prevotella* RC9 gut group, and *Rhodococcus* ↑ *Butyricimonas*, *Oscillospira*, and *Sutterella* ↓	Li and Zhao, 2015	454 sequencing
	Gammaproteobacteria ↑	Porphyromonadaceae ↑	*Escherichia Shigella* ↑	Li et al., 2016b	454 sequencing
	Profecbaciena gamma subdivision ↑		*Atopobium*, *Coriobacterium*, and *Bifidobacterium* ↓	Kleessen et al., 2005	
		Ruminococcaceae ↓	*Prevotella* and Lachnospiraseae ↓, *Faecalibacterium*, *Bacteroides*, and *Bifidobacterium* ↑	Lan et al., 2017	MiSeq sequencing
Patients with coronary heart disease	Bacteroidetes, Dialiste ↓, Firmicutes ↑		*Blautia* and *Succinivibrio* ↑	Liu F. et al., 2020	16S rDNA
Patients with liver cirrhosis	Barnesiella, Peptococcus, Melainabacteria ↓	Peptococcaceae, Succinivibrionaceae ↓, Streptococcaceae ↑	*Succinivibrio* ↓, *Prevotella*, *Streptococcus*↑	Huan et al., 2021	16S rRNA
Rats			*Peptostreptococcus*, *Bifidobacterium*, *Bacteroides*, *Lactobacillus*, and *Clostridium* ↑	Adak et al., 2014	
			*Parabacteroides*, *Alistipes*, and *Lactococcus* ↑	Pan et al., 2022	
Plateau pika			*Prevotella*, *Ruminococcus*, and *Treponema* ↑,*Oscillospira* ↓	Li et al., 2016a	16S rRNA
Martes zibellina			*Lactobacillus* ↓, *Pseudomonas* ↑	Su et al., 2021	
Rhesus macaques	Firmicutes ↑, Bacteroidetes ↓			Wu et al., 2020	16S rRNA
Tibetan antelope	Firmicutes ↑	Ruminococcaceae ↑		Ma et al., 2019	16S rRNA

High altitude can also enhance the side effects of some drugs and further increase the health risk of patients with basic diseases, such as diabetes, cerebrovascular diseases, and angina pectoris (Sun et al., 2020).

The plateau environment applies a certain selective pressure to the intestinal flora. In an analysis of shared genes between human plateau populations and plateau pigs, it was found that 80.5% of the bacterial genes were shared between them, which exceeds the highest degree of gene sharing among the intestinal microflora of humans (Zeng et al., 2020). The α- and β-diversity of the intestinal flora in the plateau population is considerably higher than that in the plain population, and the abundance of most genera in the intestines of the plateau population is higher than that of the plain population. Moreover, *Weissella*, *Clostridium*, *Butyricicoccus*, *Parasutterella*, and *Klebsiella* were more abundant in the plain population than in the plateau population (Li et al., 2016c; Liang et al., 2021). The intestinal flora of rhesus monkeys in high-altitude environments is mainly composed of *Sclerotinia* and *Rumen cocci*, whereas in low-altitude environments, this flora mainly comprises *Bacteroides* and *Prevotella*; the ratio of Firmicutes/Bacteroidetes in high-altitude environments is three times higher than that in low altitude environments (Wu et al., 2020).

There are more bacteria-producing short-chain fatty acids (SCFAs) in the host intestine in high-altitude environments. The abundance of *Archaea*, *Prevotella, Holdemanella*, and *Methanobrevibacter* in the intestinal tract of the Qinghai–Tibet plateau population is high (Liang et al., 2021). After comparing the intestinal flora of the plateau and plain populations through 454 pyrosequencing, Li and Zhao (2015) found that the abundance of *Sclerotinia* in the intestine of the plateau population was notably higher than that of the plain population, whereas the abundance of *Bacteroides* was lower than that in the plain population. One study found that the plateau population was rich in *Pseudomonas* and *Streptococcus* (Ma et al., 2021).

Some species in the intestinal tract show positive or negative correlations with altitude. One study found that Tibetans living at an altitude of 4,800 m were rich in *Clostridia*, *Clostridiales*, *Lachnospiraceae*, *Pseudobutyrivibrio*, and *Rikenellaceae*, which were also found in Tibetans living at an altitude of 3,600 m (Li et al., 2016b). In plateau pika, the abundance of *Bacteroides* in the intestinal tract is increased and the phylum Chlamydomonas decreases with elevation (Li et al., 2016a). Furthermore, the abundance of lactic acid bacteria in the intestinal tract of wild sable decreases with altitude, whereas the relative abundance of *Pseudomonas* increases with altitude (Su et al., 2021).

In some diseases, the changes in intestinal flora are different between the plateau and plain populations. The number of Bacteroidetes and *Dialister* in the intestine of patients with coronary heart disease at high altitude was less than that of healthy high-altitude population, while the number of Firmicutes, *Blautia*, and *Succinivibrio* increased (Liu F. et al., 2020). However, in plain patients, *Collinsella* population increased, whereas *Rothia* and *Eubacterium* spp. populations decreased (Karlsson et al., 2012). *Clostridiaceae _ 1, Clostridium _ sensu _ stricto _ 1, Barnesiella, Peptocucus, Ambiguous _ Taxa, Gastran Aerophiles, Peptocococaceae, Melainabacteria, Succinivibrio, Succinivionaceae*, and *Aeromonaales* were markedly decreased in the intestinal tract of high-altitude patients with liver cirrhosis; however, *Prevotella _ 2, Streptococcus, Streptococcaceae, Lactobacilles*, and *Bacilli* were increased (Huan et al., 2021). Intestinal *Bacteroides* and *Lachnospiraceae* decreased in the plain cirrhosis population, whereas *Proteobacteria*, *Fusarium* spp., *Enterbacteriaceae*, *Veillonellaceae*, and *Streptococcaceae* increased (Chen et al., 2011).

The abundance of Prevotella was positively correlated with the severity of high-altitude reaction. The severity of AMS was also associated with higher fecal microbiota diversity 1 week after AMS onset. Body weight loss at high altitudes was associated with lower relative abundances of *Lactobacillus* and *Turicibacter* (Karl et al., 2018).

## Mechanism underlying the plateau environment-mediated alteration of the host intestinal flora

### TLR4 signaling pathways and inflammatory mediators

**FIGURE 1:**
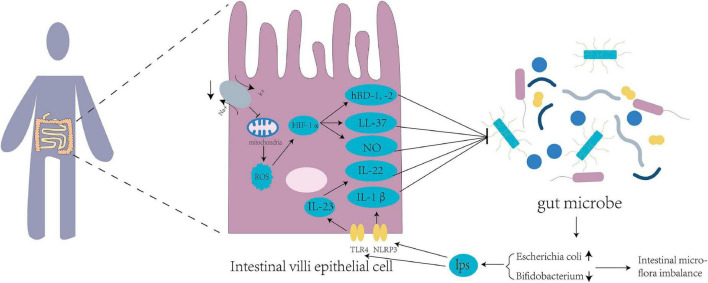
Tl R4 signaling pathways and inflammatory mediators disturb the intestinal flora of the host. Mitochondrial function is inhibited by suppression of sodium and potassium pumps at high altitude, leading to excessive production of reactive oxygen species (ROS) in the mitochondria and increased expression of hypoxia-inducible factor 1α (HIF-1α), thus producing nitric oxide (NO) and antibacterial peptides HBD-1, HBD-2, and LL-37. Intestines at high altitude produce excessive lipopolysaccharides (LPS) into the blood circulation, which increases the expression of TLR4 and NLRP3 in the intestinal epithelial cells. TLR4 upregulates the expression of IL-23 and downstream target IL-22, while NLRP3 upregulates the expression of IL-1β. These factors affect the intestinal flora of populations living in Qinghai-Tibet plateau.

For humans entering the plateau for the first time, the environment can influence the imbalance in intestinal flora through a variety of mechanisms (Figure 1). Hypoxia alters the energy pathways and mitochondrial function of cells, resulting in autoxidation and a decrease in their ability to utilize oxygen, leading to the excessive production of reactive oxygen species (ROS) (Loiacono and Shapiro, 2010). Excessive ROS levels lead to oxidative stress in the body, reducing intestinal flora diversity, promoting specific bacterial growth, and aggravating intestinal floral imbalance (Weiss and Hennet, 2017). In addition, both ROS and hypoxic conditions can lead to the accumulation of hypoxia-inducible factor-1α (HIF-1α), which can increase the expression of human β-defensin-1, -2 (hBD-1, -2), and human cathelicidin-related antimicrobial peptide (LL-37). The increase in these antimicrobial peptides further affects the intestinal flora of the host (Shao et al., 2018). HIF-1α can increase the expression level of nitric oxide synthase (Lu et al., 2006). The synthesis and release of low NO levels can protect the intestinal mucosa (Kubes, 1993). In contrast, high NO levels can cause intestinal floral disorder, promote the reproduction of pathogenic bacteria, reduce the abundance of probiotics, and damage intestinal function (Leitao et al., 2011).

High-altitude environments increase the abundance of gram-negative bacteria in the human intestinal tract and the decomposition of lipopolysaccharide (Han et al., 2021). Lipopolysaccharides, also known as endotoxins, enter the blood circulation from the damaged intestinal mucosa and stimulate the release of inflammatory mediators of different cell types (Maldonado et al., 2016), resulting in increased expression of TLR4 and NLRP3 in small intestinal cells (Xu et al., 2014; Qiu et al., 2021). The high-altitude environment may affect the intestinal microbiota by regulating TLR4/NF-κB signaling (Liu P. et al., 2021). TLR4 upregulates the expression of IL-23 (Kang et al., 2018), and IL-23 and its downstream target IL-22 are involved in the regulation of the intestinal flora (Fatkhullina et al., 2018). IL-22 controls the production of antimicrobial peptides and can be utilized by pathogens to inhibit the growth of competing bacteria, thereby improving pathogen colonization on the mucosal surface (Behnsen et al., 2014). The intestinal NLRP3 inflammasomes increase the expression of the downstream effector molecule IL-1β (Yao et al., 2017). IL-1β has been shown to play an important role in regulating the production of antimicrobial peptides (Hu et al., 2015) to regulate the intestinal flora.

### Intestinal flora regulates blood pressure and energy metabolism

**FIGURE 2:**
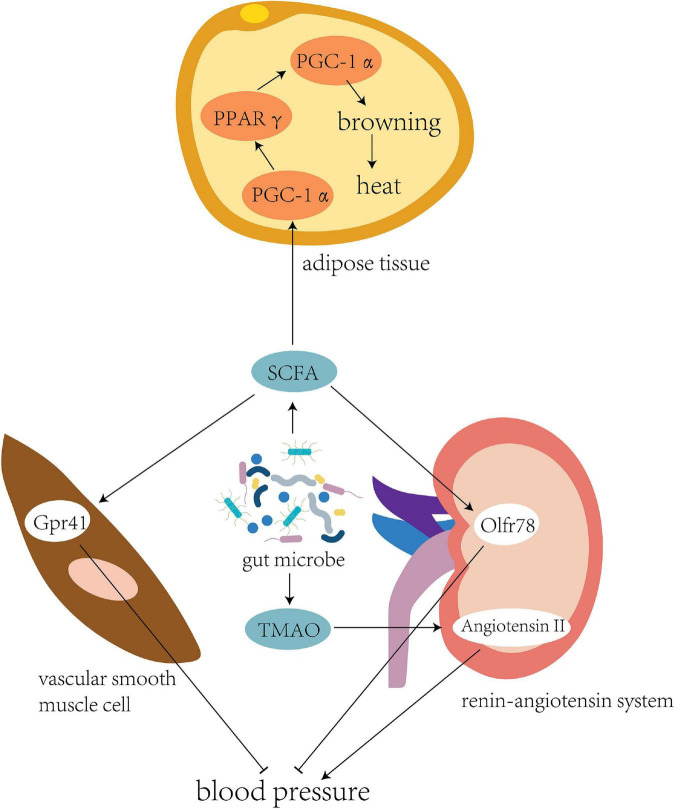
Intestinal flora regulates blood pressure and energy metabolism. The intestinal flora of individuals living in the plateau environment can produce a large number of short-chain fatty acids to provide energy. This promotes the expression of transcription cofactor PGC-1α, upregulates the peroxisome proliferator-activated receptor (PPARγ), and mediates the coupling protein UCP1, thereby inducing browning of the white fat to generate more heat to adapt to the cold plateau environment. Meanwhile, binding of SCFA to the Gpr41 and Olfr78 receptors could effectively regulate blood pressure in the high-altitude population. Another bacterial product, TMAO, could directly inhibit the pressor effect of angiotensin II to fight against elevated blood pressure caused by high altitude.

The intestinal flora has flexibility and plasticity to adapt to changes, a variety of environments, and adverse factors (Zmora et al., 2019; Figure 2). The cold and hypoxic environment of the Qinghai–Tibet plateau can seriously affect the energy balance of the human body, where the energy requirements of its inhabitants are high (Jia et al., 2020). In the Tibetan population living on the plateau, the abundance of bacteria producing SCFAs in the intestinal tract is high (Li et al., 2016b). Cecal samples of wild house mice collected from different altitudes were subjected to 16S rRNA sequencing, which showed that the abundance of *Prevotella* bacteria in the intestine positively correlates with the altitude at which the mice were located (Suzuki et al., 2019). *Prevotella* can produce a variety of SCFAs, such as butyrate, to provide energy to intestinal epithelial cells.

The microbiota under cold conditions might promote white adipose tissue browning to increase host tolerance toward cold environments. The body temperature of sterile and antibiotic-treated mice was considerably lower than that of mice colonized by normal flora (Kluger et al., 1990). Mice transplanted with a microbiota adapted to cold conditions showed higher levels of brown adipose tissue markers, increased insulin sensitivity, and decreased adipocyte levels in white adipose tissue (Chevalier et al., 2015).

Brown adipose tissue (BAT) are mitochondria-rich and uncoupling protein 1 (UCP1)-rich adipocytes, and a high UCP1 level can efficiently convert chemical energy into heat (Rosen and Spiegelman, 2014). Brown adipose cells are dominated by the sympathetic nervous system, which enables BAT to respond to stimulation, such as the cold environment of the Qinghai–Tibet plateau, and circulate locally generated heat to other parts of the body (Bartness and Ryu, 2015). SCFAs can be used as modulators of lipogenesis in the development and differentiation of brown adipocytes (Hu et al., 2016). The transcription cofactor peroxisome proliferator-activated receptor-gamma coactivator (PGC)-1α is a key molecule that stimulates the differentiation of brown adipocytes and interacts with peroxisome proliferator-activated receptor γ (PPARγ) to directly participate in the transcription of lipogenic genes (Puigserver et al., 1998). PGC-1α also interacts with PPARγ and other nuclear hormone receptors in brown adipose cells to upregulate the expression of brown adipose-specific UCP1 (Cassard-Doulcier et al., 1994; Barbera et al., 2001). After acetate treatment, PGC-1α expression increases in brown adipose cells, together with mitochondrial mass and UCP1 expression, suggesting that acetic acid induces the browning of adipose tissues (Hu et al., 2016).

Blood pressure regulation is one of the key physiological responses of humans and other animals that allows them to adapt to high-altitude environments, and sympathetic nervous system stimulation induced by hypoxia can lead to a continuous increase in blood pressure (Wolfel et al., 1994; Ivy and Scott, 2015). Bilo et al. (2019) studied 47 healthy volunteers and found a positive correlation between human blood pressure, elevated altitude, and exposure time at high altitudes (Bilo et al., 2019). The intestinal flora may be involved in the regulation of blood pressure in high-altitude populations.

Li et al. (2017) performed fecal transplantation from hypertensive individuals to germ-free mice. As the microbiota shifted, blood pressure also increased in these mice, suggesting a role for the gut microbiota in hypertension. Pluznick et al. (2013) and Pluznick (2014) found that SCFA-mediated activation of Olfr78, a metabolite of gut bacteria, can lead to increased blood pressure, while propionic acid-mediated Gpr41 activation can lead to a decrease in blood pressure. Another metabolite, trimethylamine N-oxide, has been shown to delay the vasopressor-boosting effect of angiotensin II (Ufnal et al., 2014).

## Conclusion and future perspectives

The relationship between various diseases and the intestinal flora has become a recent hot topic, but research on the effect of Qinghai–Tibet plateau on the host intestinal flora is slightly lagging. The interaction between the host and intestinal flora in a plateau environment is complex. A plateau environment can create an imbalance in the intestinal flora, which helps the host adapt by regulating energy metabolism and blood pressure. The TLR4 signaling pathway may play an important role in this process. However, the mechanisms are evidently complex, and more research is needed to elucidate them. Future research should also aim to determine the function of various bacteria in the adaptation of people to the conditions on the Qinghai–Tibet plateau. Furthermore, there has been little research on the association between the intestinal flora and diseases under plateau conditions. The application of procedures to alter the intestinal flora, such as probiotics and fecal bacteria transplantation, to help with adapting to high-altitude conditions and to fight against high-altitude diseases may be the highlight of future research. However, owing to the severity of high-altitude diseases and the complexity of the intestinal flora, researchers still face challenges. This review could provide a framework for further research, allowing future studies to focus on utilizing the relationship between the intestinal flora and high-altitude environments, which will help address these challenges.

## Author contributions

All authors made significant contributions to the conception and design, participated in the drafting of the article and in the critical revision of the intellectual content, and approved the final manuscript.
